# Highly Uniform and Porous Polyurea Microspheres: Clean and Easy Preparation by Interface Polymerization, Palladium Incorporation, and High Catalytic Performance for Dye Degradation

**DOI:** 10.3389/fchem.2019.00314

**Published:** 2019-05-08

**Authors:** Muhammad Sohail Bashir, Xubao Jiang, Shusheng Li, Xiang Zheng Kong

**Affiliations:** College of Chemistry and Chemical Engineering, University of Jinan, Jinan, China

**Keywords:** uniform microspheres, porous polyurea, interfacial polymerization, palladium incorporation, dye degradation

## Abstract

Owing to their high specific surface area and low density, porous polymer materials are of great importance in a vast variety of applications, particularly as supports for enzymes and transition metals. Herein, highly uniform and porous polyurea microspheres (PPM), with size between 200 and 500 μm, are prepared by interfacial polymerization of toluene diisocyanate (TDI) in water through a simple microfluidic device composed of two tube lines, in one of which TDI is flowing and merged to the other with flowing aqueous phase, generating therefore TDI droplets at merging. The polymerization starts in the tube while flowing to the reactor and completed therein. This is a simple, easy and effective process for preparation of uniform PPM. Results demonstrate that the presence of polyvinyl alcohol in the aqueous flow is necessary to obtain uniform PPM. The size of PPM is readily adjustable by changing the polymerization conditions. In addition, palladium is incorporated in PPM to get the composite microspheres Pd@PPM, which are used as catalyst in degradation of methylene blue and rhodamine B. High performance and good reusability are demonstrated. Monodispersity, efficient dye degradation, easy recovery, and remarkable reusability make Pd@PPM a promising catalyst for dye degradation.

## Introduction

Transition-metal-based catalysts are of great importance to pharmaceutical, environmental and fine chemical industries. The majority of these catalytic metal species are toxic, making their handling potentially hazardous; and they are in general high-costing, limiting therefore their application, albeit their high performance well-known. Their “clean and easy” synthesis with reliable reusability has driven the generation of a variety of new strategies for metal incorporation and immobilization to make the catalyst easy to recover and reuse, and to keep them at an acceptable cost (Ley and Baxendale, 2002; Zhang, 2005; Paul and Das, 2012; Romanazzi et al., 2018).

Polyurea (PU) is known for long as important engineering polymer materials thanks to their thermal shock and abrasion resistance, high impact resistance, good flexibility, water repellency etc. (Awad et al., 2009; Delebecq et al., 2013). Common applications of PU have been protective coating materials for different structural materials (Davidson et al., 2005; Mohotti et al., 2013; Samiee et al., 2013), and relevant researches are being conducted to explore their novel applications (Ley et al., 2003; Koyama and Nakamura, 2010; Leventis et al., 2010; Cass et al., 2013; Li et al., 2013; Weigold et al., 2013; Yadav and Lawate, 2013; Han et al., 2016; Jiang et al., 2017). Conventional synthesis of PU materials has been commonly done through step growth polymerization using diisocyanate and diamines (Zhu et al., 2006; Feng and Iroh, 2013), use of multifunctional amines or isocyanates is also reported (Moon et al., 2010; Kim et al., 2011). It is well-known that isocyanate (NCO) is highly reactive toward water, turning itself into amine by release of CO_2_. The *in-situ* formed amine groups copolymerize quickly with NCO groups, leading to PU formation (Leventis et al., 2010; Jiang et al., 2012, 2017; Li et al., 2013; Weigold et al., 2013; Han et al., 2016). Obviously, this process of PU synthesis is advantageous because the reactions involve only isocyanate monomer and water; it is a more cost effective and environment friendly approach than the classic synthesis of PU, because it replaces expensive amines with water, and in general it proceeds readily at ambient temperature. Through this process, two types of PU materials of different morphologies have been prepared, i.e., solid microspheres without porous structure (Jiang et al., 2012, 2017), and porous PU without regular form or shape (Leventis et al., 2010; Li et al., 2013, 2015a; Weigold et al., 2013; Han et al., 2014, 2016). It is easy to conceive that solid microspheres are of low specific surface area, and their use as support for enzyme and transition metal is not suitable because the amount of immobilized reagent on their surface are limited, or inaccessible as catalytic active site if incorporated in the microspheres; whereas for porous materials with irregular forms, it makes their recovery difficult due to their irregularity combined with their size difference, particularly for the very small ones. It is therefore highly interesting to prepare porous and uniform polymer microspheres as the support for transition-metal-based catalysts and enzyme immobilization (Wu et al., 2012; Petkovich and Stein, 2013). Up to date, PU microspheres were prepared only with isophorone diisocyanate (IPDI) through precipitation polymerization (Jiang et al., 2012, 2017); with toluene diisocyanate (TDI), porous PU of irregular form was always obtained because of the chain rigidity of the resulting PU (Han et al., 2014; Li et al., 2015a).

We report here a simple and effective process for preparation of highly uniform and porous PU microspheres (PPM) through interfacial polymerization of TDI with the aid of a simple microfluidic device, composed of two tubing lines, adopted with modification from published reports (Quevedo et al., 2005; Lee et al., 2008; Perez et al., 2015; Petit et al., 2017). Simply by dissolving Pd(OAc)_2_ in TDI, PPM with palladium (Pd) encapsulation (Pd@PPM) is easily fabricated. The hybrid composite, Pd@PPM, is used for degradation of two organic dyes. High catalytic activity with full reusability is observed within 10 recycled uses. This work provides therefore a facile pathway to the fabrications of highly uniform and porous PU with Pd incorporation, Pd@PPM, which is featured not only by its clean and easy preparation through a novel strategy, but also by its high performance as catalyst for dyes degradation, combined with its facile recovery and reusability with sustainable catalytic activity.

## Experimental Section

### Materials

All chemicals were China domestic. Toluene diisocyanate (TDI, industrial grade, a mixture of 2,6- and 2,4-isomers of 20/80), from Beijing Keju Chemicals Ltd.; Polyvinyl alcohol (PVA-0588, industrial grade, degree of polymerization of 500 with 88% of hydrolysis), of Kaidu Industrial Co., Ltd, Shanghai; Acetone (AP grade) from Laiyang Fine Chemicals, Shandong; Rhodamine B (RhB), sodium borohydride (NaBH_4_, AR) and palladium acetate, Pd(OAc)_2_, (AR, Pd 46–48%) from Aladdin Biochemical Technology Co. Ltd, Shanghai; Methylene blue (MB) from Damao Chemicals, Tianjin. Ultra-pure water was obtained from Millipore instrument and used throughout the process.

### Microfluidic Device Design for Preparation of Uniform PPM

**Figure 1:**
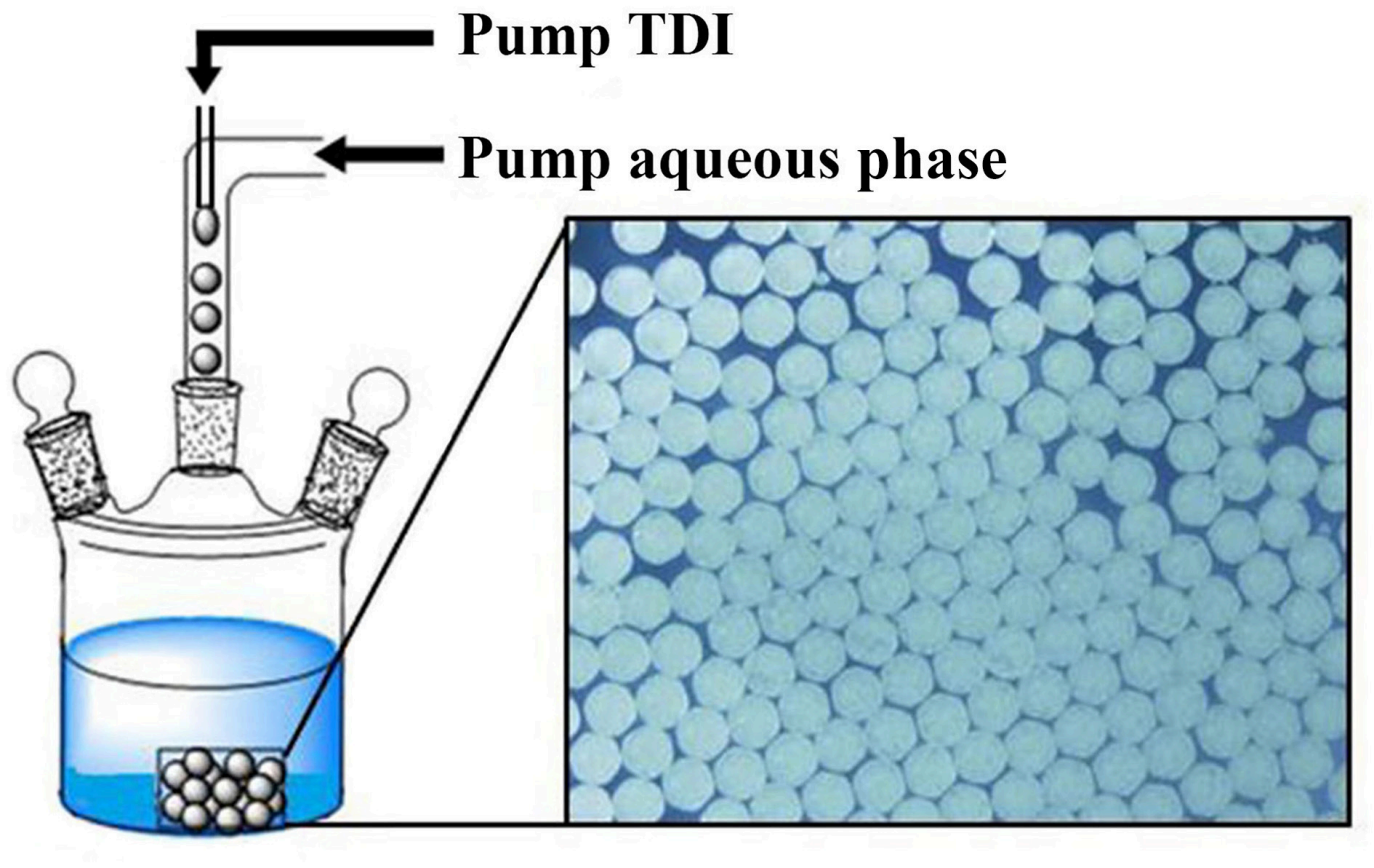
Systematic diagram of preparation process for uniform PPM.

To assure the formation of uniform microspheres, a simple microfluidic device was used, inspired by similar devices reported (Quevedo et al., 2005; Lee et al., 2008; Perez et al., 2015; Petit et al., 2017), which is composed of two tube lines. In one tube is flowing aqueous PVA solution and in the other TDI monomer. The tube of aqueous flow is connected with one end to a pump (TS2-60, from Longer Precision Pump Co., Ltd.) and the other to the reactor. Silicone tube of 0.3 mm for the inner diameter (Shenchen Rubber Plastic, Shanghai) was used for both flows. For TDI flow, a syringe needle (30 G, interior diameter of 159 μm) was used at end of the tube, and pierced in the tube of the aqueous flow, so that TDI flow was merged into the aqueous flow as depicted in Figure 1. Individually separated TDI droplets were formed in the aqueous phase and flowed into the reactor at a desired temperature. Except otherwise stated, the flow rate was fixed at 2.5 mL/min for the aqueous phase and at 40 μL/min for TDI. TDI droplets were allowed to continue their conversion to PU in the reactor. At end of polymerization, the microspheres were collected by filtration and dried under vacuum.

### Incorporation of Pd in PPM and Catalytic Activity of Pd@PPM in Dye Degradation

To incorporate Pd in the PPM, exactly the same process was used except that 90 mg of Pd(OAc)_2_ was dissolved in TDI (6.5 g) by ultra-sonication prior to charging TDI into its syringe, and the Pd(OAc)_2_ solution in TDI was introduced into the aqueous flow in the tubing system. At end of the polymerization, light brown PPM with Pd incorporated (Pd@PPM) were obtained. Pd@PPM was used as catalyst for MB degradation in the presence of NaBH_4_ as the reducer. A typical run is described as following: MB (5 mL, 50 μM) and fresh prepared NaBH_4_ solution (0.5 mL, 0.2 M) were located into a bottle of 10 mL capacity at room temperature, followed by addition of Pd@PPM catalyst (0.05 g, containing 0.035 mg of Pd) under gentle stirring in order to get Pd@PPM well dispersed in the reaction system. After a time interval, the reaction was stopped by halting stirring and the bottle reactor left standing on bench top. The Pd@PPM microspheres were settled down quickly at the bottle bottom, the supernatant was separated out simply with transfer pipette, poured into quartz cuvette and subjected to UV-vis analysis to determine the residual MB concentration by measuring its absorbance at 664 nm. The same degradation was carried out for RhB, except that the absorbance at 554 nm was used to determine its residual concentration. Dye degradation with respect to time was also determined by halting the degradation reaction at different time. The degradation efficiency was calculated by Equation (1) below:

(1) $$\text{Degradation efficiency }(\%)=(1-\;C/C_{0})\;\times 100\%$$

Where, *C*_0_ is the initial concentration of the dye, and *C* the concentration at time *t*.

### Characterization of PPM and Pd@PPM

The sizes and shape of TDI droplets, the microspheres PPM and Pd@PPM were examined under optical microscope (OM, BX-51, Olympus). Their size (D_n_) and size distribution (D_w_/D_n_) were obtained by counting at least 200 microspheres. Surface and inner morphology of the microspheres were observed under scanning electronic microscope (SEM, Quanta FEG-250, FEI). Their porous property was examined by BET (Nora 200E, Quantachrome) and mercury intrusion porosimetry (AutoPore IV 9500, Micromeritics). In addition, Pd@PPM was also examined using Inductive Coupled Plasma Optical Emission Spectrometer (ICP-OES, Optima 5300DV, Perkin-Elmer) and EDS (X-Max50, Oxford) analysis to affirm the presence of Pd. Temperature-programmed reduction (TPR) and chemisorption were performed using AutoChem II 2920 for the measurement of Pd dispersion. Powder X-ray diffraction (XRD) was done on a diffractometer (D8 Focus, Bruker).

## Results and Discussion

### PU Formation Mechanism

The chemical reactions involved here are quite simple (see Figure S1): TDI reacts with water to turn its NCO groups into carbamic acid, which is unstable and turns to the corresponding primary amine with the release of CO_2_ (Jiang et al., 2012; Han et al., 2014). Because the reactivity of TDI toward amines is much higher than toward water (Delebecq et al., 2013; Han et al., 2014; Jiang et al., 2017), there occurs the step-growth polymerization of TDI with the *in-situ* formed amines, leading to PU. Through this step-growth polymerization via water, we have prepared highly uniform PU microspheres by precipitation polymerization of isopherone diisocyanate (IPDI) in binary mixtures of water-acetone (Jiang et al., 2012) and water-acetonitrile (Jiang et al., 2016, 2017) as well as in a ternary mixture of water-acetone-DMF (Wei et al., 2019). However, with IPDI replaced by TDI, it was impossible to prepare microspheres by the same process; Granular PU with irregular form and size were always obtained, owing to the chemical structure of TDI, leading to a PU consisted of only carbamic units and aromatic rings. The chains are highly rigid and prone to precipitate out once formed. The primary particles formed can hardly keep spherical because of the rigidity of the chains, and a quick aggregation of the particles would take place (Han et al., 2014; Li et al., 2015a). In order to achieve well shaped microspheres, a microfluidic device is designed as depicted in Figure 1, based on the reported studies from different researchers (Quevedo et al., 2005; Lee et al., 2008; Perez et al., 2015; Petit et al., 2017). The device is composed of two tube lines. In one was flowing aqueous PVA solution and in the other TDI monomer. TDI flow was merged into the aqueous flow, to form individual TDI droplets in the aqueous phase and flowed into the reactor at a desired temperature. The main function of this device is to transfer the previous precipitation polymerization into interfacial polymerization while keeping the microsphere surface clean without use (or very low amount) of stabilizer or surfactant. This interfacial polymerization must be quick enough to form a hard shell on the surface of TDI droplets, so that to protect them from deformation and aggregation once flowed into the reactor. Addition of a polyamine, ethylene diamine for example, is often needed to accelerate this shell formation (Jiang et al., 2018). TDI droplets remained stable this way to keep their shape in the reactor at 60°C. Full conversion of TDI was accomplished in the reactor. TDI conversion was determined using a reported procedure (Jiang et al., 2018). The results indicated that TDI reached its final conversion at 4 h of polymerization at 60°C (Figure S2). In this study, polymerization lasted 5 h to assure the highest TDI conversion.

PU chains thus formed are consisted of phenylene (from TDI) and urea groups alternatively (Figure S1). CO_2_ liberated in this reaction was the primary foaming agent for pore generation. It is to note that the shell of the microspheres was formed at very beginning stage of the polymerization, encapsulating liquid TDI inside. This part of TDI was converted to polymer by its reaction with water at a slow rate because of the difficulty for water molecules to diffuse inside of the PU spheres with a hard shell. Pores of large and medium sizes were thus formed with the progress of the polymerization. This has been well-studied previously (Han et al., 2014; Li et al., 2015a; Jiang et al., 2018). PVA was used here as a surfactant to adjust the size of the PPM, its reaction with NCO groups is believed not occurring given its extreme low content and the presence of abundant water in the system, though the reaction, yielding polyurethane, was reportedly possible (Romaskevic et al., 2010; Strakšys et al., 2013). The possible reaction of the amide protons at both sides of the urea carbonyl group with NCO group, leading to biuret formation (Qiu et al., 2018) must not take place since this reaction was believed to occur under specific experimental conditions, such as high temperature above 130°C, NCO largely in excess or with specific catalyst (Han et al., 2014; Qiu et al., 2018). The PPM as prepared here was therefore consisted of linear polymer only.

### Effect of Flow Rates of the Aqueous Phase and TDI

**Figure 2:**
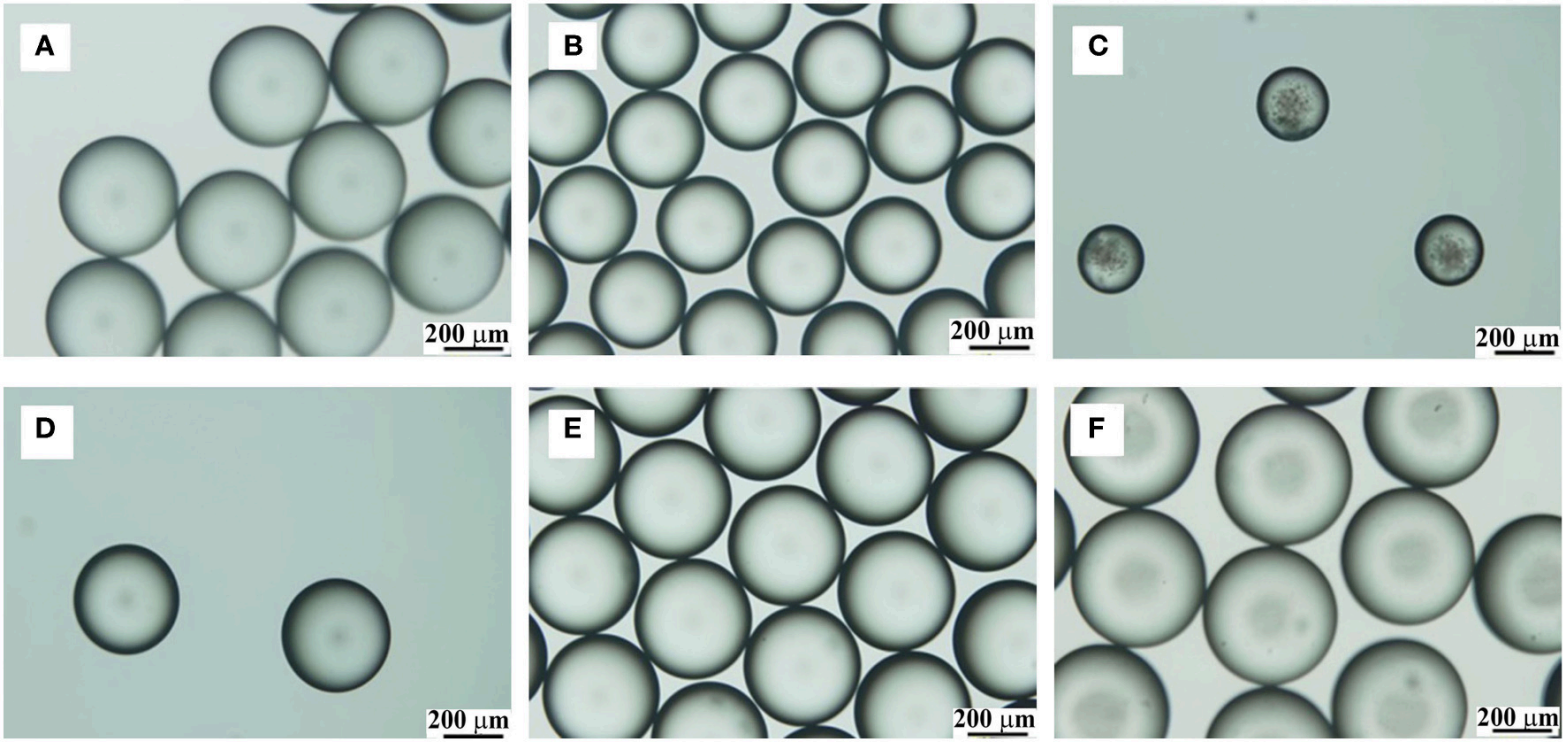
OM photos of PPM prepared with fixed TDI flow rate (20 μL/min) and varied flow rate for the aqueous phase (mL/min): 0.1, **(A)**; 2.1, **(B)**; 5.7, **(C)**. OM photos of PPM prepared with fixed flow rate for aqueous phase (1.0 mL/min) and varied TDI flow rate (μL/min): 5, **(D)**; 30, **(E)**; 150, **(F)**.

Based on the mechanism of PPM formation, TDI droplets were individually formed at exit of the syringe nozzle of TDI stream, and kept as is while flowing in the aqueous phase down to the reactor. To produce uniform microspheres, it is crucial to have a hard shell formed on the TDI droplets so that to protect them from deforming or aggregation. It is easy to understand that the flow rates of the aqueous phase and TDI must impose key influences on the PPM formation. The effects of the two flow rates were therefore first studied. TDI is practically insoluble in water. At the tip of TDI syringe nozzle just before merging into the aqueous flow, liquid TDI was subjected to different forces, including for instances, its interface tension against water, the pushing pressure from the pump behind, its shearing friction with the syringe nozzle etc. (Clanet and Lasheras, 1999; Gokmen and Du Prez, 2012; Jiang et al., 2018). With the tubing system and the flow liquids being the same, the study was initiated with varied flow rate, in such a way that TDI flow was fixed while varying that of the aqueous phase, and vice versa. In a first set of experiments, aqueous phase flow rate was changed from 0.1 to 5.7 mL/min with TDI flow rate kept at 20 μL/min. The size of PPM was determined from their OM photos shown in Figures 2A–C.

**Figure 3:**
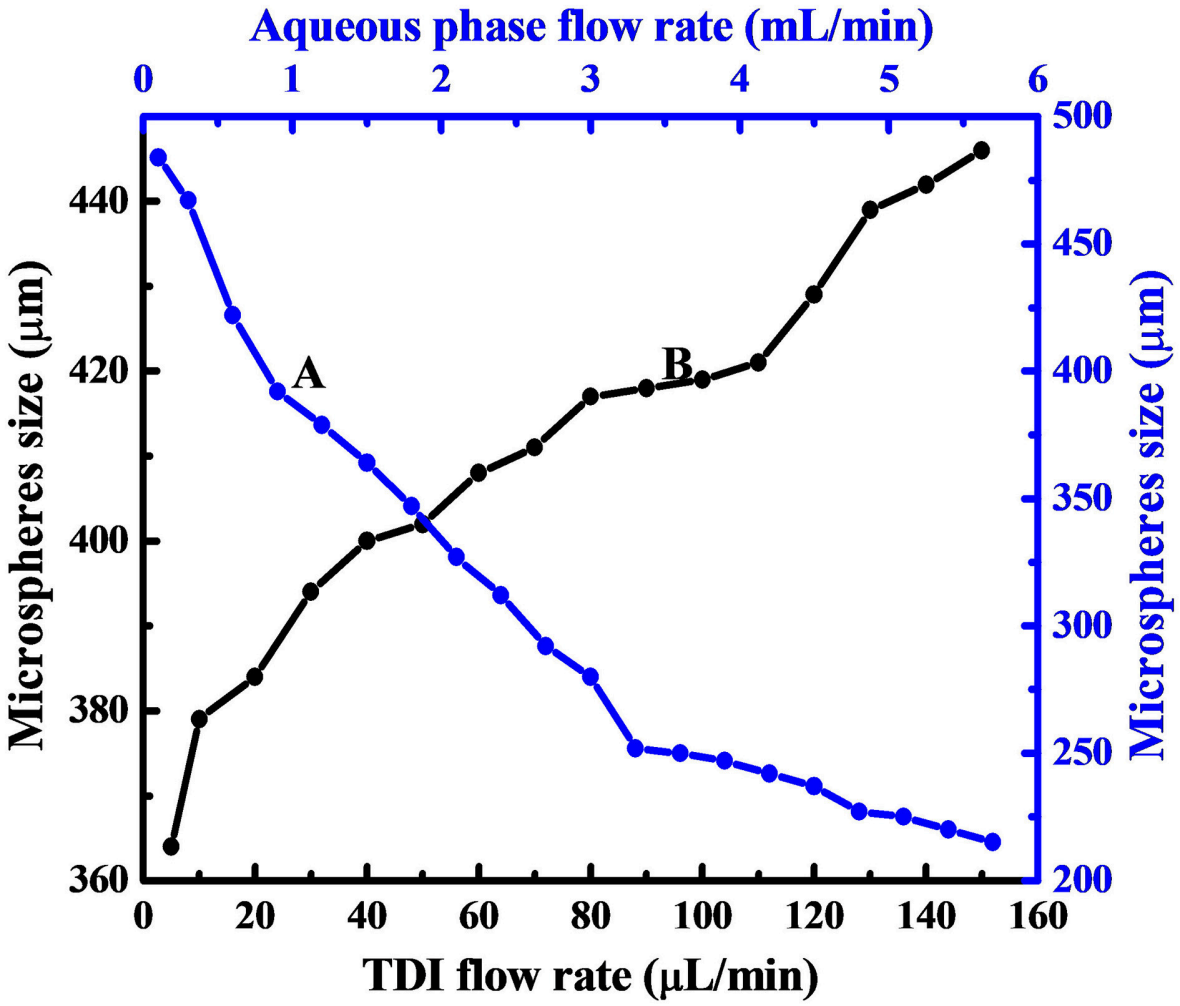
Dependence of PPM size on aqueous phase flow rate with TDI flow rate fixed at 20 μL/min **(A)**, and that on TDI flow rate with aqueous phase flow rate fixed at 1.0 mL/min **(B)**.

The size dependence on the flow rate of aqueous phase is displayed in Figure 3 by curve A, which reveals clearly that, the size of PPM regularly decreased from 484 to 215 μm by increasing aqueous flow rate from 0.1 to 5.7 mL/min. This was because the emission time between two TDI droplets to pinch off from the syringe tip was decreased with increased aqueous phase flow rate. The size of TDI droplets was then regularly reduced, so was the size of PPM at end of the polymerization. This is well seen from Figure 2, which demonstrates also that all PPM was highly uniform regardless of their sizes.

As to the influence of TDI flow rate on the size of the resulting PPM, the results given in Figure 3 (curve B) show clearly that the size of the PPM was increased from 364 to 446 μm with TDI flow rate increased from 5 to 150 μL/min. It is to note that here the flow rate of the aqueous phase was fixed (1.0 mL/min), which meant that the emission time between two TDI droplets from the syringe tip was practically constant, and that an increase in TDI flow rate gave more TDI accumulated in the time interval between two consecutive emissions of TDI droplet, the size of the individual droplet was therefore increased, so was the size of PPM at end of the polymerization. OM images of selected PPM obtained at different TDI flow rate are also shown in Figures 2D–F, which shows that all the PPM were highly uniform, and their size was in constant increase with increasing TDI flow rate as shown in Figure 3 (curve B).

### Effect of PVA Amount on PPM

**Figure 4:**
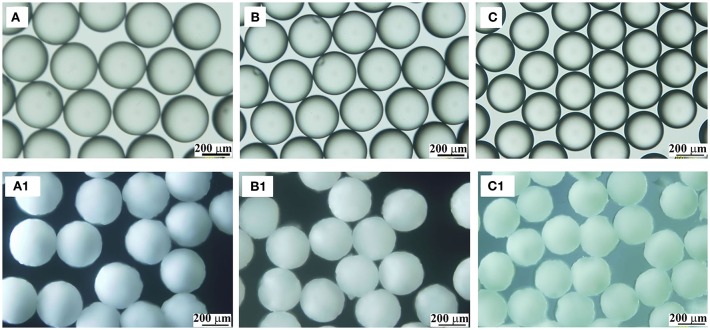
OM images of TDI droplets **(A–C)** and the corresponding polymer microspheres **(A1–C1)** prepared at 60°C with different PVA amount: 0.05 wt%, **(A,A1)**; 0.5 wt%, **(B,B1)**; 1.5 wt%, **(C,C1)**.

As aforementioned, the interface tension between liquid TDI and water must play an important role in the process, because this interface tension is also a determinate factor for the formation of TDI droplets, and therefore the size and shape of PPM (Clanet and Lasheras, 1999; Gokmen and Du Prez, 2012). The effect of PVA amount, varied from 0 to 1.5 wt% relative to water mass, was therefore explored, while the other conditions kept unchanged (flow rate of aqueous phase: 2.5 mL/min; TDI flow rate: 40 μL/min; polymerization at 60°C). In addition to the final PPM, the size of the droplets just at their entry to the reactor, assumingly with a PU shell formed, was also determined. The results are listed in Table 1. It is to point out that, without presence of PVA, TDI droplets were easily aggregated and formed large irregular lumps or grits soon after their arrival into the reactor, indicating that the PU shell formed was thin and fragile, and not hard enough to protect the droplets from packing up. As long as there was 0.05 wt% of PVA in the aqueous phase, highly uniform PPM in perfectly spherical shape were well-formed. This observation suggested that the presence of PVA was required as a supplementary assistance to keep the droplets stable. The size of TDI droplets shows that they were all slightly larger than those of PPM (Table 1; Figure 4). Knowing that the size of the droplets was determined at wet state, and that of PPM done at dried state after their separation and drying up under vacuum, the slight size difference was not unexpected.

**Table 1:** Characteristics of TDI droplets and PPM prepared with different PVA amount (Polymerization at 60°C; aqueous phase flow rate, 2.5 mL/min; TDI flow rate, 40 μL/min).

**PVA (wt%)**	**Droplet size (D_n_, μm)**	**PPM size (D_n_, μm)**	**D_w_/D_n_**
0.05	344	340	1.009
0.10	341	338	1.009
0.50	333	327	1.010
1.00	310	305	1.013
1.50	290	281	1.014

The data in Table 1 demonstrate that, with increased PVA amount, both the sizes of the droplets and PPM were decreasing. That was because PVA was functioning as surfactant (Kong et al., 2009). With increased PVA amount, the surface tension of TDI droplets was reduced, their compatibility with water increased, their detachment from syringe nozzle and moving into water phase were facilitated, and the time interval between two consecutive detachments was shortened, leading to smaller TDI droplets, and by consequence smaller sized PPM. OM photos of selected TDI droplets and PPM are given in Figure 4 for visual illustration, which shows that both the droplets and PPM were quite monodisperse regardless of PVA amount used.

### Effects of Polymerization Time and Temperature on the Formation of PPM

Based on the formation mechanism of PPM described above, the function of the microfluidic device is the formation of uniform TDI droplets in the tubing system, which gives TDI droplets the time needed to form a hard shell on their surface by interfacial polymerization, so that to endow them with a good stability against their aggregation or collision when they reach to the reactor. Based on this conception, it is easy to conceive that the size and the shape of PPM would not be affected by the polymerization time in the reactor, providing that the PU shell be formed before the droplets flowing into the reactor. This was in fact well-confirmed by the experimental results (Table S1; Figure S3), which show that the size and its distribution (D_w_/D_n_) of the PPM were practically constant from 2 to 20 h, and that the PPM thus prepared was indeed highly monodisperse with a very narrow size distribution (D_w_/D_n_), with polydispersity index of 1.009 at 3 h of polymerization, and 1.002 thereafter.

Under exactly the same experimental conditions as above, the polymerization was conducted at different temperature, varied from 30 to 80°C. The results revealed that a slight increase in the size of PPM was observed with increased temperature (Table S2; Figure S4). This slight increase in size, limited to a few microns and representing <1% with an increase of 10°C, was ascribed simply to the increase in TDI volume owing to the increased temperature of polymerization. No size was given for the PPM prepared at 30°C because the PPM were broken down in the subsequent washing and drying, most likely owing to the formation of an extremely thin shell because of the slow reaction at this low temperature. A slightly larger size distribution (D_w_/D_n_) was also seen for the samples prepared at temperature below 60°C (Table S2), indicating that highly uniform PPM was warranted only at 60°C or higher, when the polymerization was accelerated and a shell with the thickness large enough was formed to protect the microspheres from deformation or aggregation. This was also confirmed from the optical photos of PPM (Figure S4), where the PPM prepared below 60 °C appeared more or less deformed.

### Morphology Observation by SEM and Porous Property

**Figure 5:**
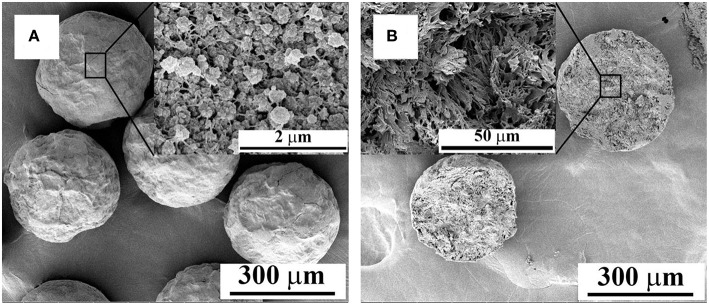
SEM images of PPM showing the surface **(A)** and interior **(B)** morphology (Sample prepared with: 0.05 wt% of PVA in aqueous phase; Flow rate of aqueous phase, 2.5 mL/min; flow rate of TDI, 40 μL/min; Polymerization temperature, 60°C).

Surface and interior morphology of PPM were examined under SEM, and selected photos given in Figure 5 show clearly a porous structure on the surface and inside of the PPM. In principal, these pores were formed by the release of CO_2_, produced in the formation of PU. Porous properties of the material, i.e., pore size distribution and specific surface area, were also obtained from BET and MIP (Figures S5, S6). From BET test, a continuous pore size distribution was detected from 0 to 250 nm with a significant concentration around 20 nm; whereas from MIP test, two main pore size distributions were seen, one at 400 nm with broad pore size distribution from 50 nm to 4 μm, and another large distribution around 100 μm. These tests confirmed that there were not only large pores as seen from SEM photos, but also small pores with size below 50 nm, as detected by BET test. The specific surface area was 28.0 m^2^/g from BET, with a pore volume of 0.047 cm^3^/g; and only 5.2 m^2^/g was detected for the specific surface area by MIP, with a porosity of 68%. These data demonstrate that PPM were typically porous materials consisted of large and small pores.

**Figure 6:**
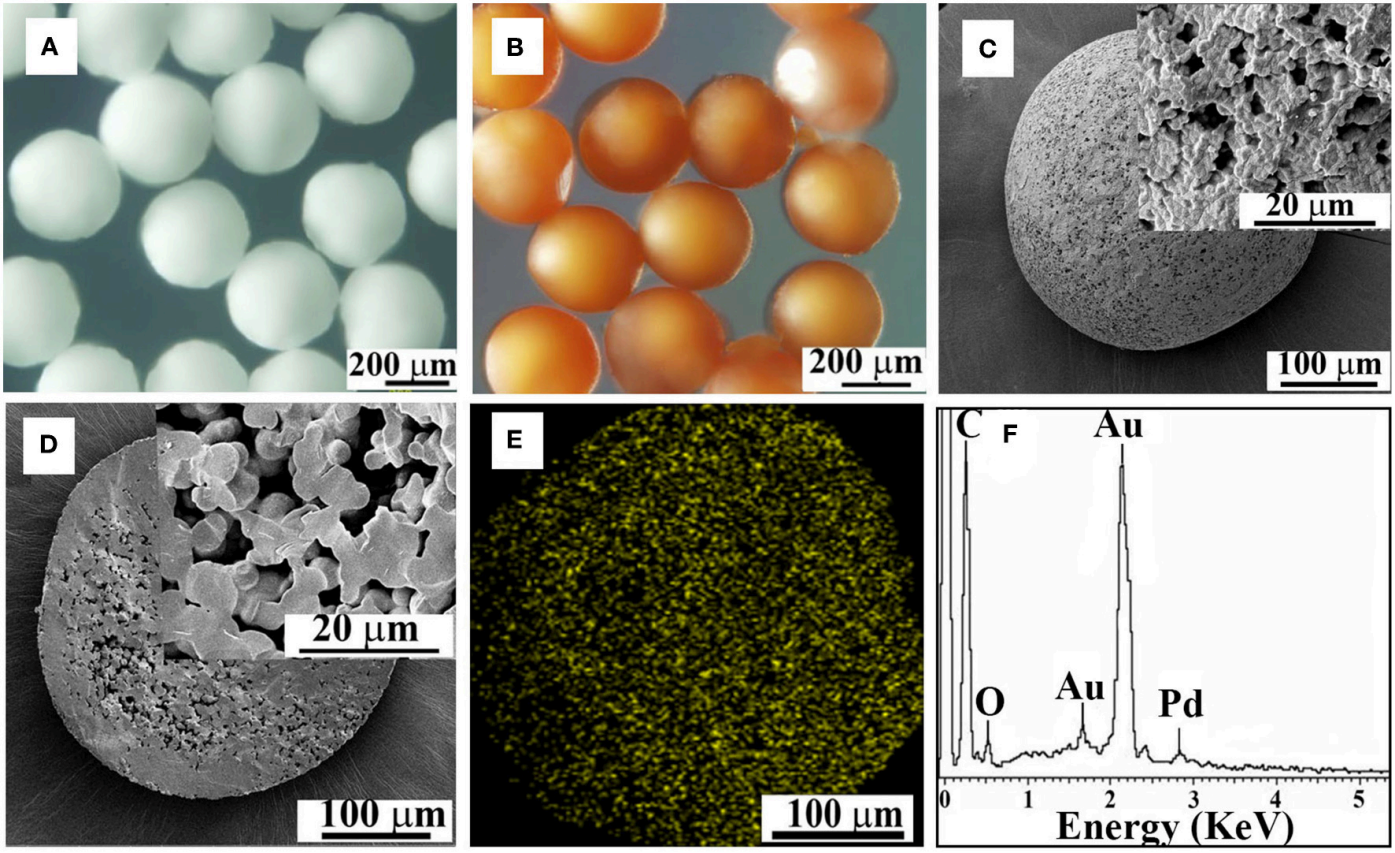
OM images of PPM without **(A)** and with Pd incorporation (**B**, Pd@PPM), SEM images of the surface **(C)** and interior structure **(D)** of Pd@PPM, EDS mapping and spectrum of Pd@PPM **(E,F)**.

PPM incorporated with Pd (Pd@PPM) was also subjected to OM and SEM examinations for morphology observation. Pd@PPM (Figure 6B) was seen to possess exactly the same morphology and uniformity as PPM, the microspheres without Pd incorporation (Figure 6A). From SEM pictures (Figures 6C,D), it is seen that Pd@PPM catalyst is also of porous structure. EDS analysis showed clearly Pd presence (Figures 6E,F), thanks to PU chemical structure, and particularly its urea groups in PPM, which can ligate and therefore retain Pd metal (Ramarao et al., 2002). ICP-OES test gave 0.07 wt% of Pd in Pd@PPM, which was also confirmed by pyrolysis of Pd@PPM samples at 500°C for 7 h. XRD test on Pd@PPM showed diffraction peaks similar to PPM, indicating that the structure of PPM (Han et al., 2014) was not changed upon Pd loading. However, independent diffraction peak of Pd was not observed in the diffractogram of Pd@PPM (Figure S7), owing to most likely the extreme low Pd content. To identify the active sites on Pd@PPM, CO pulse chemisorption analysis was performed. By assuming stoichiometry of CO/Pd being unity (Mahata and Vishwanathan, 2000), 41.2% of Pd dispersion was detected with their active diameter of about 27.06 nm combined with metallic surface area of 18.44 m^2^/g.

### Catalytic Property of Pd@PPM in Dye Degradation

**Figure 7:**
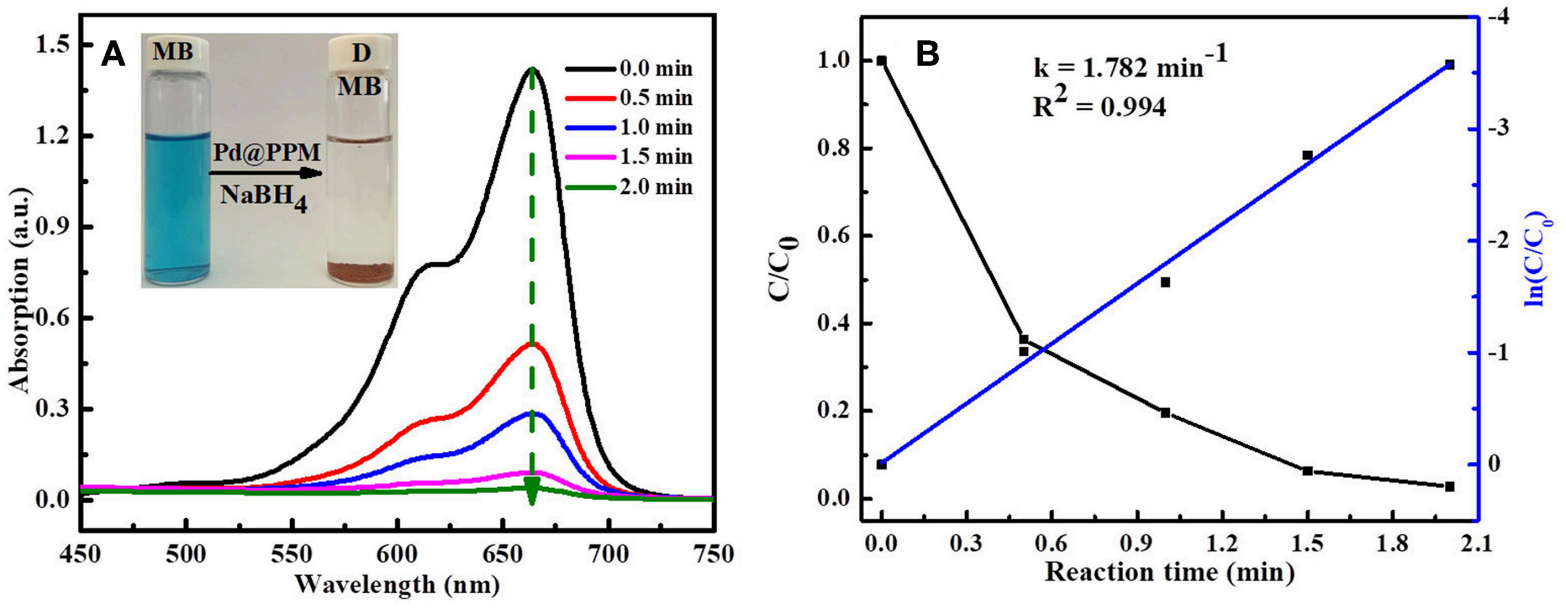
Evolution of UV-vis absorption in MB (5 mL, 50 μM) degradation: **(A)** using Pd@PPM (50 mg) and NaBH_4_ (0.5 mL, 0.2 M); **(B)** the corresponding degradation kinetics.

To study the catalytic activity of Pd@PPM, degradation of MB was tested. MB (5 mL, 50 μM) and fresh prepared NaBH_4_ solution (0.5 mL, 0.2 M) were located into a bottle of 10 mL capacity, followed by addition of Pd@PPM catalyst (50 mg, containing 0.035 mg of Pd). Concentration of the residual MB was determined at different degradation time, and the results are presented in Figure 7A, which demonstrated that Pd@PPM was indeed a highly effective catalyst for MB degradation, a full degradation was achieved at about 2 min, where the initially blue MB solution turned very clear, with the brown Pd@PPM catalyst settled at bottom of the bottle within 2 min after stirring halted (Figure 7A, inset). Two parallel tests were carried out, one with Pd@PPM only without NaBH_4_ and another with NaBH_4_ only without Pd@PPM under otherwise exactly the same experimental conditions. The results revealed that the degradation did not occur (Figures S8A,B), which suggests that Pd@PPM is highly effective as the catalyst only in the presence of NaBH_4_, none of them alone is effective for MB degradation.

The reaction kinetics of MB degradation was studied by following the intensity of UV-vis spectra at fixed time interval, based on a run done with MB (5 mL, 50 μM), Pd@PPM (50 mg, containing 0.035 mg of Pd) in the presence of NaBH_4_ (0.5 mL, 0.2 M) at 25°C. By calculation of MB concentration ratio (*C/C*_0_), obtained from the relative absorption peaks ratio (*A/A*_0_) at different time, it was observed that dye degradation reaction followed the pseudo-first-order reaction kinetics (Equation 2)

(2) $$ln(C/C_{0})\;=\;ln(A/A_{0})\;=\;-k\times t$$

Where, *C* is the MB concentration at time *t, C*_0_ the initial MB concentration, *A* the absorption peak at time *t*, and *A*_0_ that at start of the degradation (*t* = 0). *k* is the reaction rate constant and calculated by plotting ln(*C/C*_0_) vs. reaction time *t*. The slope of straight line is the reaction rate constant and its value was 1.782 min^−1^ as shown in Figure 7B.

### Effect of Pd@PPM and NaBH_4_ Amount on Dyes Degradation

**Figure 8:**
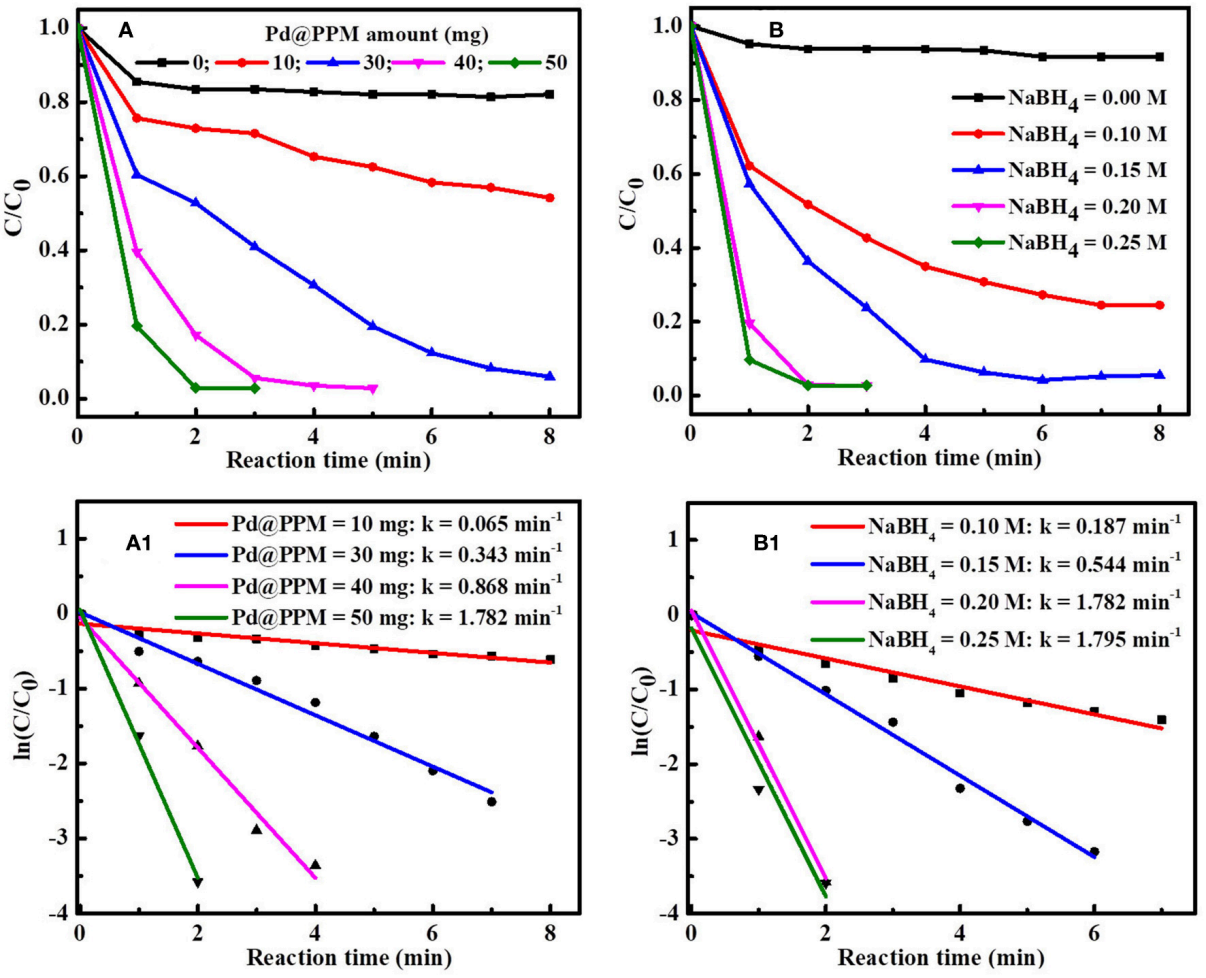
Effect of Pd@PPM amount on MB (5 mL, 50 μM) degradation **(A)** and the rate constant (k) obtained with different Pd@PPM amount and fixed NaBH_4_ amount (0.5 mL, 0.2 M) **(A1)**; Effect of NaBH_4_ amount on MB (5 mL, 50 μM) degradation **(B)** and the corresponding rate constant (k) obtained with different NaBH_4_ amount and fixed Pd@PPM amount (50 mg, containing 0.035 mg of Pd) **(B1)**.

The effect of Pd@PPM amount on MB degradation was studied by keeping NaBH_4_ (0.5 mL, 0.2 M) and MB (5 mL, 50 μM) constant. The results are shown in Figure 8A. It is observed that, with increase in Pd@PPM amount, MB degradation was accelerated. With 30 mg of Pd@PPM, more than 97% of MB was degraded within 7 min; while MB was practically full degraded (>99.6%) within 2 min with 50 mg of Pd@PPM. At the same time, the rate constant of MB degradation was increased in accordance with accelerated degradation, as shown by the results in Figure 8A1. The observed higher degradation rate with higher amount of Pd@PPM is easy to understand because the number of the active sites is certainly increased with increased amount of Pd@PPM, which in turn facilitates the contact of the dye molecules with ${\text{BH}}_{4}^{-}$ ions on catalyst surface, making the degradation accelerated.

Similarly, effect of NaBH_4_ amount on the degradation was also studied, with fixed Pd@PPM (50 mg) and MB amounts (5 mL, 50 μM) (Figures 8B,B1). It is seen that MB degradation was enhanced with increase in NaBH_4_ amount when NaBH_4_ concentration was below 0.2 M, and this enhancement was significant attenuated with NaBH_4_ reaching to 0.2 M, corresponding to a molar ratio of about 304 for NaBH_4_/Pd (See Figure S9). This indicates that an excessive amount of NaBH_4_ against Pd was necessary to reach a high performance for the degradation, as often observed also in reported studies (Fu et al., 2013; Li et al., 2015b; Zhang et al., 2016).

To further evaluate the catalytic activity of Pd@PPM, the turn over frequency (TOF, expressed in mol_MB_/mol_cat_/min, based on the total mass of Pd) was calculated, which gave 0.379 mol_MB_/mol_cat_/min, which is practically the same (0.380 mol/mol/min) as that reported on unsupported Pd-TNPs (Fu et al., 2013), indicating that Pd catalytic activity was fully retained in the present Pd@PPM.

The same degradation was also done with 2.0 mL of RhB aqueous solution (15 mg/L), 0.3 mL of NaBH_4_ (0.25 M) and Pd@PPM (100 mg, 0.07 mg of Pd). The results demonstrate that this Pd@PPM catalyst was also highly effective for RhB degradation. 96.6% of RhB was degraded around 1 min (see Figure S10). This is a significantly higher performance in comparison to a number of reported studies on Pd-based catalysts (Ghaedi et al., 2012; Fu et al., 2013; Li et al., 2015b) (see Table S3).

**Figure 9:**
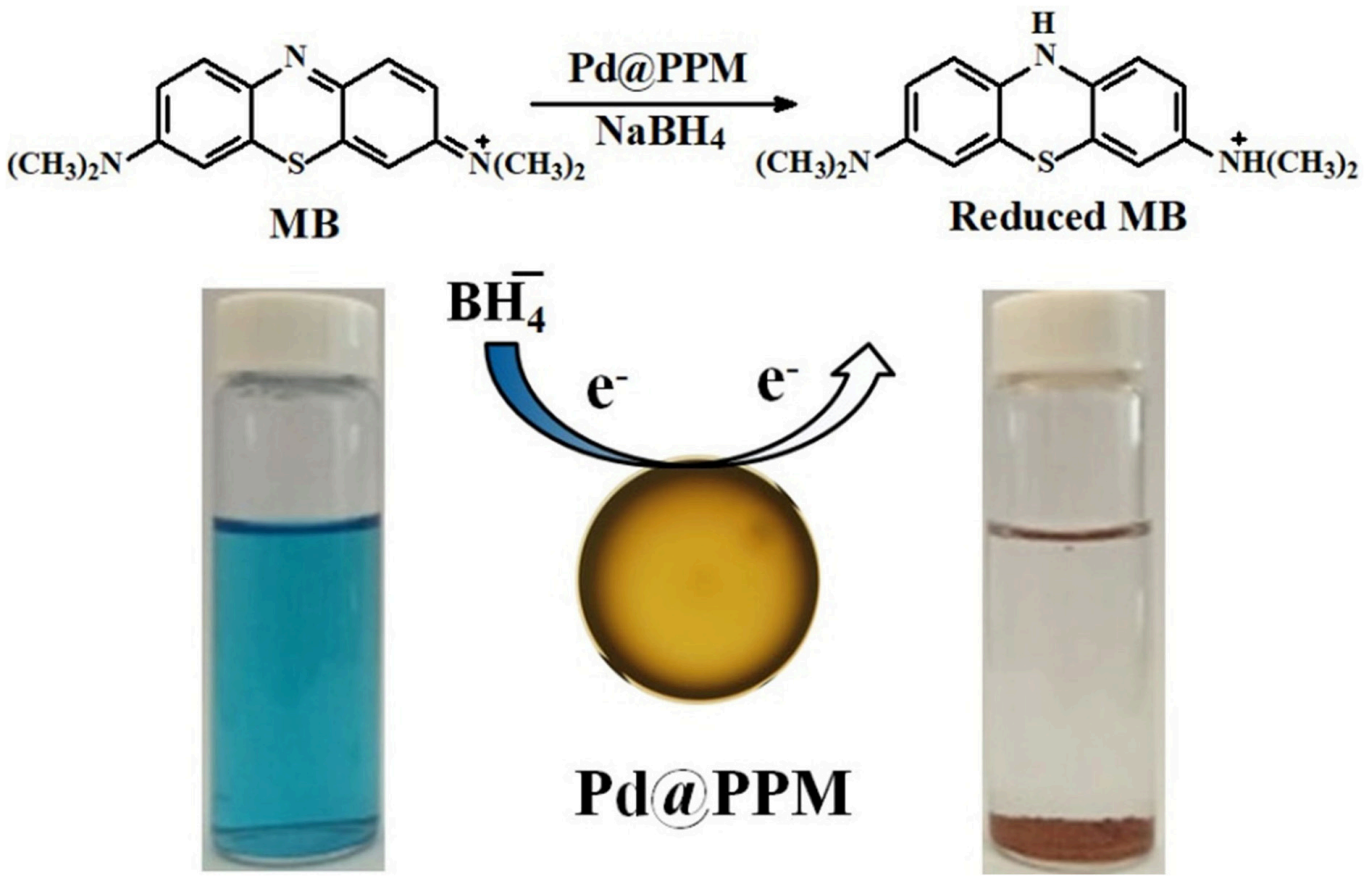
Proposed catalytic mechanism for MB degradation by Pd@PPM.

As often reported and confirmed here again in this study, the presence of NaBH_4_ is necessary for dye degradation to take place. It is known that MB is electrophilic and ${\text{BH}}_{4}^{-}$nucleophilic in nature (Sultan et al., 2018). Pd@PPM is acting as mediator and provides the surface for the adsorptions of the dye molecules and ${\text{BH}}_{4}^{-}$ anions. The function of Pd is to assist to transfer electron from ${\text{BH}}_{4}^{-}$ anions (donor) to the dye molecules (accepter), leading to dye degradation through a redox mechanism. The catalysis reaction occurs on the points where Pd is available on the surface of Pd@PPM. Therefore, the performance of the catalyst is increased with the increase in the availability of Pd on the surface of Pd@PPM. The Pd dispersion in Pd@PPM is 41.2%. As shown above, Pd@PPM catalyst is of porous structure (Figures 5, 6C,D), which was certainly beneficial to the availability of Pd, and to the effectiveness of catalysis by consequence. Moreover, the conjugation site present on Pd@PPM must be also helpful to accelerate electron transfer from ${\text{BH}}_{4}^{-}$ to dye molecules, enhancing therefore the degradation (Sultan et al., 2018). The dye molecules, once degraded, were desorbed from the surface of the catalyst, providing therefore a released free surface for the reaction to continue (Fu et al., 2013; Zhang et al., 2014; Li et al., 2015b). The general mechanism for catalytic degradation of dye molecules is schematized in Figure 9.

### Reusability of Pd@PPM Catalyst

The recoverability and reusability are the important criteria to judge a supported catalyst. It is to note that Pd@PPM catalyst, because of the large size of the microspheres, is readily recoverable from the reaction mixture by quick sedimentation at bottom of the reactor in 2 min, driven only by gravity. The upper layer liquid became clear water (see Figure 7A, Figure S10, Photo insets). The catalyst at the end of the degradation was recovered and reused for a subsequent degradation. The results show that this catalyst has retained practically their initial activity at the 10th cycle of reuse (see Figure S11). However, the catalytic activity started to decline slightly in a perceptible manner, or a same degradation could be achieved with slightly but visibly extended catalysis time. For example, at 10th cycle of reuse in degradation of MB under the conditions given in Figure 7, about 99% of MB was degraded within 2.0 min of catalysis; at 12th cycle of reuse, about 98% of MB was degraded within the time; while at 14th recycled use, about 95.2% of MB was degraded if the catalysis time was strictly controlled at exactly 2.0 min, or a full degradation was achieved with an extended reaction time of about 2.15 min. This may indicate most likely that the amount of Pd coordinated on surface of PPM was decreasing after each use, and this decrease was minimal that it was detectable after 10 recycled uses, though further study is required to have a full understanding. This reusability out-performed most of the reported supported metal based catalysts, for which the catalytic activity was reduced more or less after as low as 4 cycles of reuses (Ghaedi et al., 2012; Fu et al., 2013; Kora and Rastogi, 2016). Taking into accounts the easy preparation of Pd@PPM and their spherical form with large size, which render Pd@PPM easy to be recovered for reuse, it is concluded that this study provides a novel process for the preparation of Pd-based catalyst of great interest and potential for applications as catalyst in dye degradations.

## Conclusions

To prepare highly uniform and porous polyurea microspheres (PPM), a simple microfluidic device was used, in which TDI droplets were generated by injecting a TDI flow into a flowing aqueous phase inside a silicone tube leading to a batch reactor. TDI conversion to polyurea started in the tube while flowing to the reactor and completed there. Using this assembled system, PPM with diameters between 200 and 500 μm were prepared by interfacial polymerization of TDI with water in the presence of PVA. Studies of the effects of PVA concentration in the aqueous phase, flow rates of the aqueous and TDI phases revealed that uniform PPM was obtained only with PVA of 0.05 wt% or higher, and PPM size was decreased with increase in PVA amount used. The size of PPM was also easily adjustable by changing either the flow rate of the aqueous phase or that of TDI independently. PPM was characterized and confirmed to be typical porous microspheres containing both large (up to several hundreds of microns) and small pores (down to several nanometers). Pd acetate was incorporated in PPM to get uniform Pd@PPM composite, which was used as catalyst for degradation of MB and RhB in the presence of NaBH_4_. This work provides therefore a facile pathway to the fabrication of highly uniform and porous PU microspheres and those with palladium incorporation, Pd@PPM. Pd@PPM was demonstrated to be high catalytic for MB and RhB degradations with sustainable reusability of 100% with 10 recycled uses.

## Author Contributions

MB designed the microfluidic device and performed the experiments. XJ and SL contributed to arranging the materials and characterization analysis. XK supervised the research and contributed to the manuscript writing. All authors read and approved the final manuscript.

### Conflict of Interest Statement

The authors declare that the research was conducted in the absence of any commercial or financial relationships that could be construed as a potential conflict of interest.
