# Supplementary material for: Highly Uniform and Porous Polyurea Microspheres: Clean and Easy Preparation by Interface Polymerization, Palladium Incorporation, and High Catalytic Performance for Dye Degradation
Source: Front Chem. 2019 May 8;7:314. doi: 10.3389/fchem.2019.00314 (PMC6518977; doi:10.3389/fchem.2019.00314)
Supplement: Supplementary file 1 [file Data_Sheet_1.PDF]

Supplementary Material

Highly uniform and porous polyurea microspheres: clean and easy preparation by interface polymerization, palladium incorporation and high catalytic performance for dye degradation

Muhammad Sohail Bashir, Xubao Jiang, Shusheng Li, and Xiang Zheng Kong*

College of Chemistry and Chemical Engineering, University of Jinan, Jinan, China

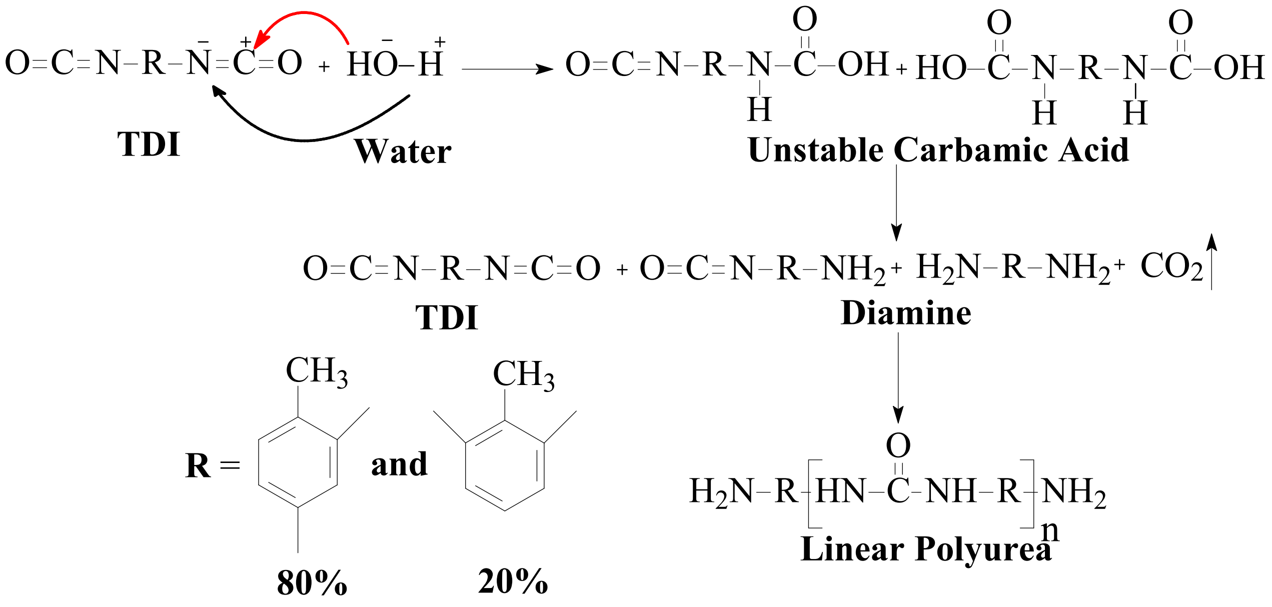

FIGURE S1. Reactions involved in polyurea formation through TDI reaction with water

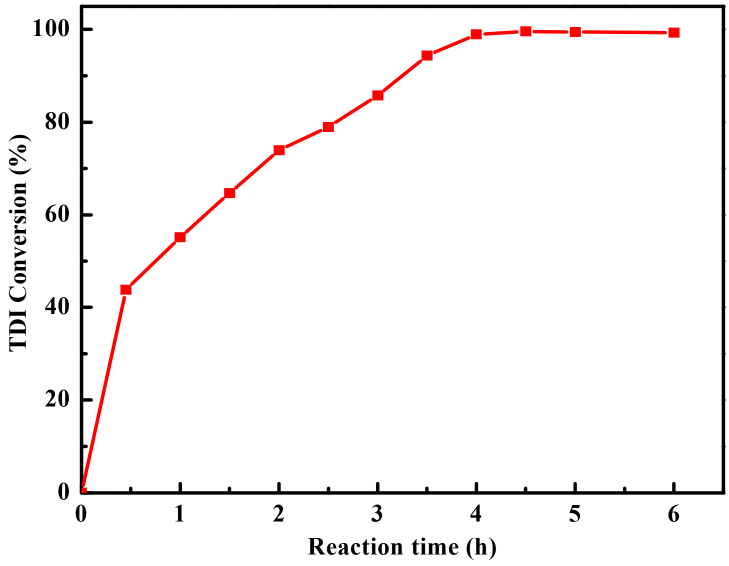

FIGURE S2. TDI conversion versus polymerization time

TABLE S1. Size and size distribution of PPM prepared at different reaction time

Polymerization Time (h)[a]	PPM size (D_n, μm)	Size distribution (D_w/D_n)
1[b]	*	*
2	340	1.009
3	340	1.009
4	341	1.002
6	342	1.002
20	342	1.002

a) Process conditions: Polymerization at 60 ˚C; aqueous phase (0.05 wt% of PVA) flow rate, 2.5 mL/min; TDI flow rate, 40 μL/min. b). Microspheres were fragile and broken down during washing and drying.

TABLE S2. Size and size distribution of PPM prepared at different temperature

Temperature (°C)	PPM size (D_n, μm)	Size distribution (D_w/D_n)
30	*	*
50	332	1.024
55	335	1.015
60	340	1.009
65	340	1.005
70	342	1.002
80	346	1.002

Process conditions: Flow rate of aqueous phase (with 0.05 wt% of PVA), 2.5 mL/min; TDI flow rate, 40 μL/min.

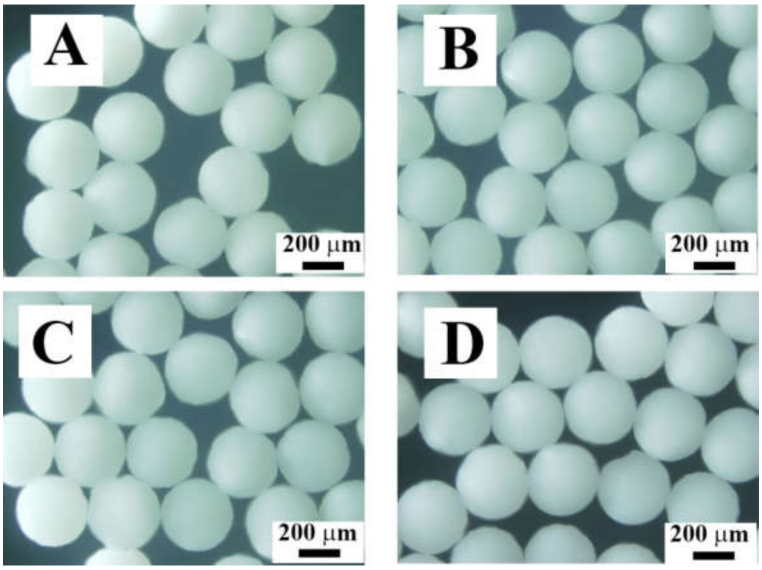

FIGURE S3. OM photos of the microspheres taken at different polymerization time (**A**, 2 h; **B**, 4 h; **C**, 6 h; **D**, 20 h). Experimental conditions: PVA in aqueous phase, 0.05 wt%; Flow rate of aqueous phase, 2.5 mL/min; TDI flow rate, 40 μL/min; Polymerization temperature, 60 °C

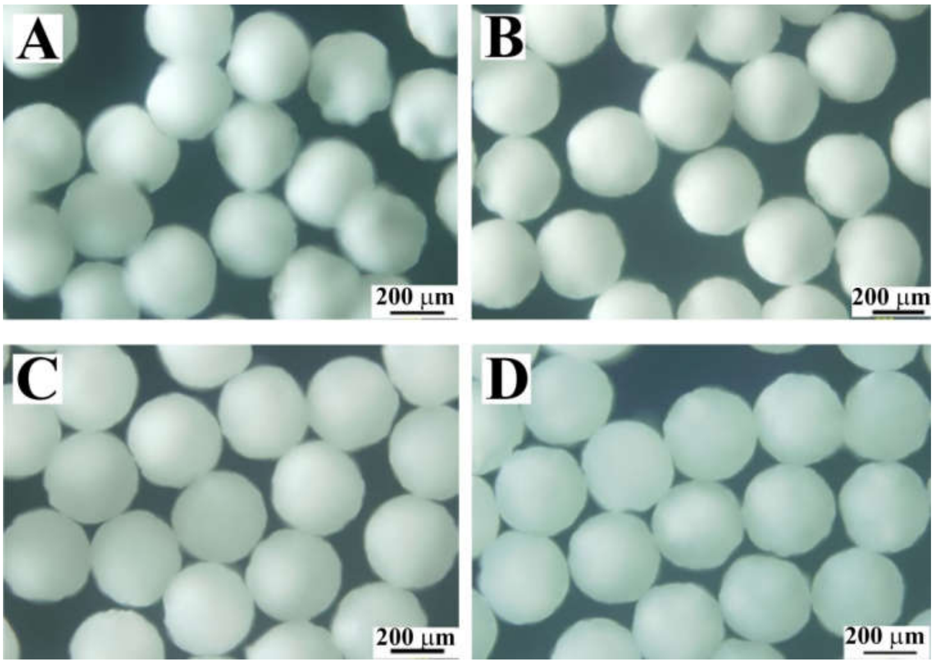

FIGURE S4. OM photos of PPM prepared at different temperature (**A**, 50 ºC; **B**, 60 ºC; **C**, 70 ºC; **D**, 80 ºC). Flow rate of aqueous phase (with 0.05 wt% of PVA), 2.5 mL /min; TDI flow rate, 40 μL /min.

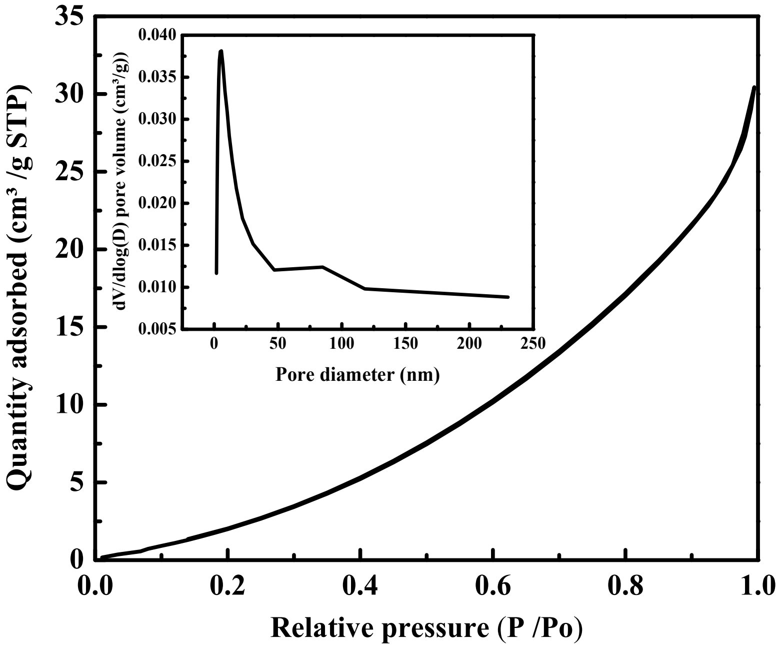

FIGURE S5. Pore size distribution and pore volume determined by BET for PPM prepared at 60 ˚C with 0.05 wt % of PVA in aqueous phase.

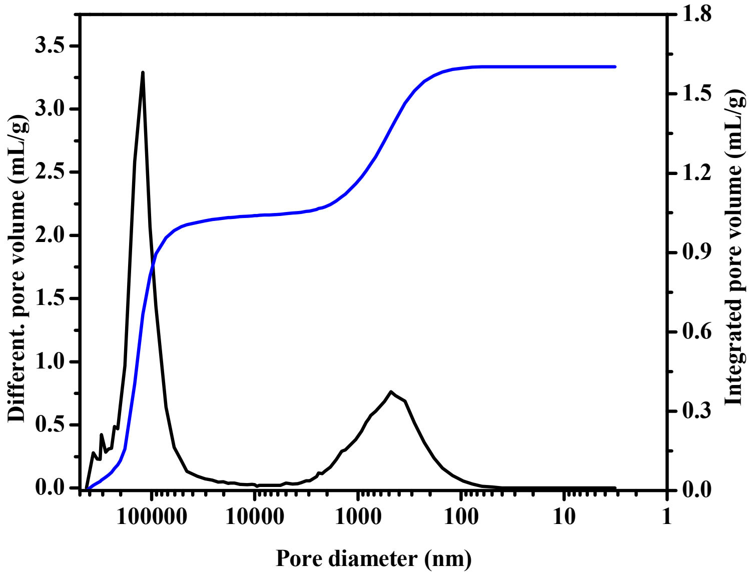

FIGURE S6. Pore size distribution and pore volume determined by mercury intrusion for PPM prepared at 60 ˚C with 0.05 wt % of PVA in aqueous phase.

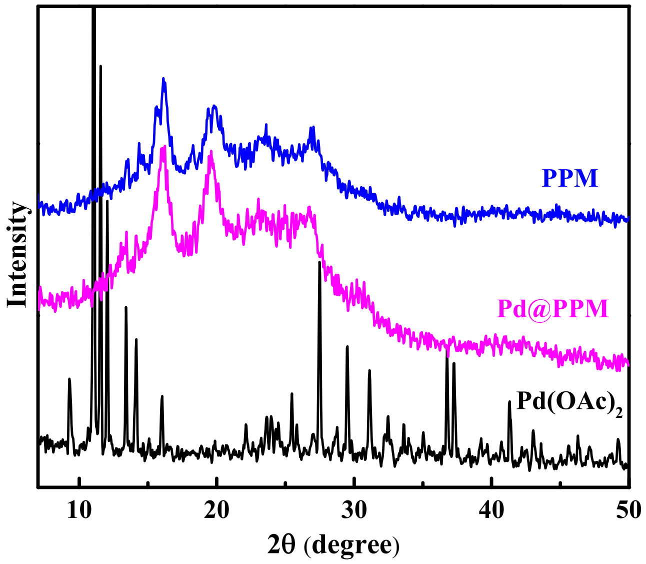

FIGURE S7. XRD diffractogram of Pd@PPM, $Pd(OAc)_2$ and PPM

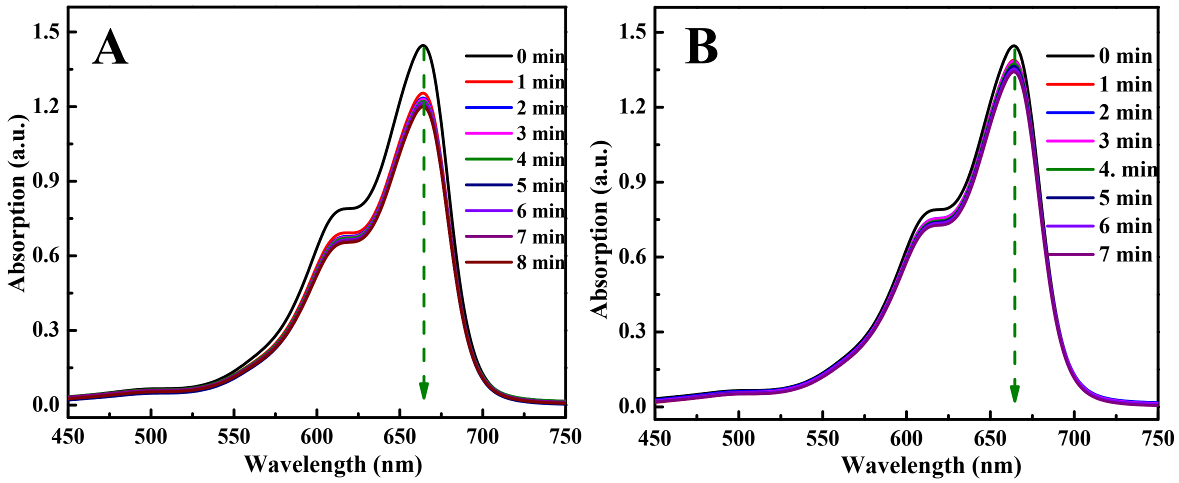

FIGURE S8. Evolution of UV-vis absorption in MB (5 mL, 50 μM) degradation using Pd@PPM (50 mg) only (A), and using $NaBH_4$ (0.5 mL, 0.2 M) only (B)

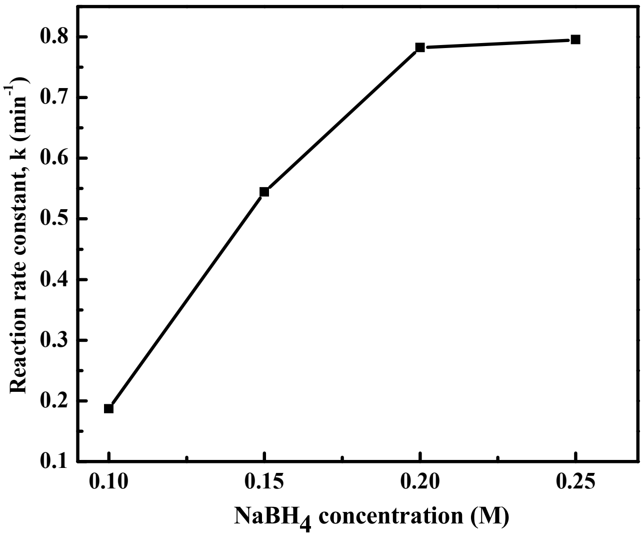

FIGURE S9. MB (5 mL, 50 μM) degradation rate constant determined using Pd@PPM (50 mg, 3.5 μmol of Pd) combined with $NaBH_4$ of different concentration.

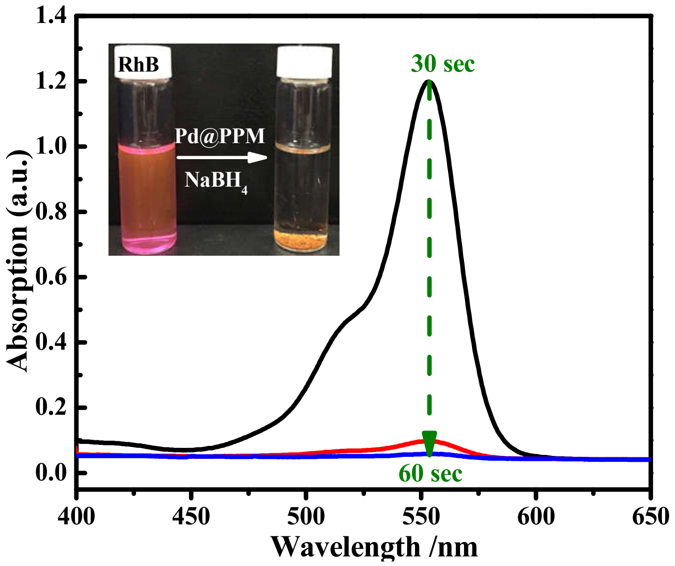

FIGURE S10. UV-vis absorption spectra for degradation of RhB (2 mL, 15 mg/L) using Pd@PPM (100 mg, containing 7 μmol of Pd) and $NaBH_4$ (0.3 mL, 0.25 M).

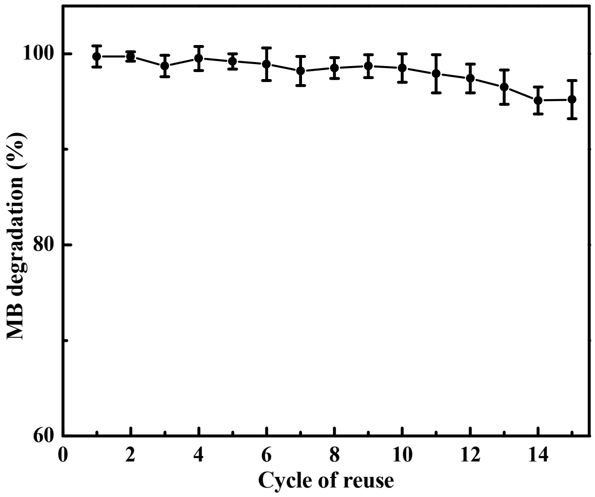

FIGURE S11. Reusability of Pd@PPM catalyst for MB degradation

TABLE S3. Performance comparison of Palladium based support material in dye degradation

Metal	Supports	Dye	Concentration (mg /L)	Removal efficiency (%)	Time (min)	Rate constant k (min^{-1})	Ref.
Pd	AC	BR	20	97%	19	0.286	Ghaedi et al., 2013
Pd	AC	MB	7	98%	9.5	0.0021	Ghaedi et al., 2012
Pd	MCN	MO	20	99.3%	60	NA	Cano et al., 2017
Pd	HOP/Fe_3O_4	MR	7	> 90%	80	0.0378	Safavi and Momeni, 2012
Pd	HOP/Fe_3O_4	CR	25	> 90%	24	0.138	Ghaedi et al., 2012
Pd-NP	NA	RhB	3.2	> 95%	2	NA	Kora and Rastogi, 2016
Pd-TNPs	RGO	MB	16	>99%	6	0.400	Fu et al., 2013
Pd	AC	CR	15	80%	18	0.130	Ahmadi et al., 2015
Pd	Fe_3O_4-PEI-RGO	MB	16	>99 %	6	0.442	Li et al., 2015
Pd	Fe_3O_4@ P(DVB/MAA)	RhB	15	99.6%	1.5	NA	Zhang et al., 2016
Pd	PPM	MB	15	99.6%	2	1.782	This work
Pd	PPM	RhB	15	99.6%	1.0	-	This work

AC, Activated carbon; HOP, Hydroxyapatite; MCN, Multiwalled carbon nanotubes; NP, Nanoparticles ; DVB/MMA, Divinylbenzene/Methacrylic acid; Pd-TNPs, tetrahedral Pd nanocrystals; RGO, reduced graphene oxide; PPM, Porous polyurea microspheres

REFERENCES CITED:

Ahmadi, K., Ghaedi, M., and Ansari, A. (2015). Comparison of nickel doped zinc sulfide and/or palladium nanoparticleloaded on activated carbon as efficient adsorbents for kinetic

andequilibrium study of removal of congo red dye. *Spectrochim Acta A. Mol. Biomol. Spectrosc*. 136, 1441-9.

Fu, G., Tao, L., Zhang, M., Chen, Y., Tang, Y., Lin, J., and Lu, T. (2013). One-pot, water-based and high-yield synthesis of tetrahedral palladium nanocrystal decorated graphene. *Nanoscale* 5, 8007–8014.

Cano, O. A., González, C. A. R., Paz, J. F. H., Madrid, P. A., Casillas, P. E. G., Hernández, A. L. M., and Pérez, C. A. M. (2017). Catalytic activity of palladium nanocubes/multiwalled carbon nanotubes structures for methyl orange dye removal. *Catal. Today* 282, 168–173.

Ghaedi, M., Ghayedi, M., Kokhdan, S. N., Sahraei, R., and Daneshfar, A. (2013). Palladium, silver, and zinc oxide nanoparticles loaded on activated carbonas adsorbent for removal of bromophenol red from aqueous solution. *J. Ind. Eng. Chem.* 19, 1209–1217.

Ghaedi, M., Heidarpour, S., Kokhdan, S. N., Sahraie, R., Daneshfar, A., and Brazesh, B. (2012). Comparison of silver and palladium nanoparticles loaded on activated carbon for efficient removal of Methylene blue: Kinetic and isotherm study of removal process. *Powder Technol.* 228, 18–25.

Ghaedi, M., Biyareh M. N., Kokhdan, S. N., Shamsaldini, S., Sahraei, R., Daneshfar, A., Shahriyar, S. (2012). Comparison of the efficiency of palladium and silver nanoparticles loaded on activated carbon and zinc oxide nanorods loaded on activated carbon as new adsorbents for removal of Congo red from aqueous solution: Kinetic and isotherm study. *Mater. Sci. Eng. C32*, 725–734.

Kora, A. J., and Rastogi, L. (2016). Catalytic degradation of anthropogenic dye pollutants using palladium nanoparticles synthesized by gum olibanum, aglucurono arabinogalactan biopolymer. *Ind. Crops Prod.* 81, 1–10.

Li, S., Li, H., Liu, J., Zhang, H., Yang, Y., Yang, Z., Wang, L., and Wang, B. (2015). Highly efficient degradation of organic dyes by palladium nanoparticles decorated on 2D magnetic reduced graphene oxide nano sheets. *Dalton Trans.* 44, 9193–9199.

Safavi, A., and Momeni, S. (2012). Highly efficient degradation of azo dyes by Pd/hydroxyapatite/Fe_3O_4 nanocatalyst. *J. Hazard. Mater*. 201-202, 125–31.

Zhang, B., Wang, J., Chen, J., Li, H., Wang, H., and Zhang, H. (2016). Fe_3O_4@P(DVB/MAA)/Pd composite microspheres: Preparation and catalytic degradation performance. *RSC Adv*. 6, 100598–100604.
